# Effect of lactobacillus reuteri-derived probiotic nano-formulation on recurrent aphthous stomatitis: a double-blinded randomized clinical trial

**DOI:** 10.1186/s12903-023-03756-8

**Published:** 2023-12-19

**Authors:** Nazafarin Samiraninezhad, Hojat Kazemi, Mostafa Rezaee, Ahmad Gholami

**Affiliations:** 1 Student Research Committee, Shiraz University of Medical Sciences, Shiraz, Iran; 2 Department of Oral and Maxillofacial Medicine, School of Dentistry, Shiraz University of Medical Sciences, Shiraz, Iran; 3 Pharmaceutical Sciences Research Center, Shiraz University of Medical Sciences, Shiraz, Iran; 4 Department of Medical Nanotechnology, School of Advanced Medical Science and Technology, Shiraz University of Medical Sciences, Shiraz, Iran; 5 Oral and Dental Disease Research Center, School of Dentistry, Shiraz University of Medical Sciences, Shiraz, Iran

**Keywords:** *Lactobacillus*, *Limosilactobacillus reuteri*, Probiotics, Stomatitis, Aphthous, Chitosan, Nanogels

## Abstract

**Objectives:**

We aimed to assess the therapeutic effects of a topical probiotic nano-formulation derived from *Lactobacillus reuteri* on treating recurrent aphthous stomatitis.

**Materials and methods:**

60 participants were randomly allocated into two groups (control and probiotic). Probiotic group administered topical probiotic nano-formulation three times a day for seven days. The control group administered a standard analgesic oral rinse. The size of ulcer(s) and pain severity were recorded on days 0, 3, 5, and 7 after intervention.

**Results:**

Before the intervention, the groups had no significant differences in terms of pain severity (*P*-value = 0.28) and lesion size (*P*-value = 0.24). Both groups exhibited significant reductions in pain severity and lesion size over the course of the intervention. After one week, the probiotic group had a notably larger lesion size reduction than the control group (*P*-value = 0.01). The probiotic group also showed a significantly greater reduction in pain severity than the control group (*P*-value = 0.04).

**Conclusions:**

Applying topical probiotic nano-formulation derived from *Lactobacillus reuteri* three times a day decreased lesion size and pain severity in RAS patients faster than the local analgesic oral rinse.

**Clinical relevance:**

*Lactobacillus reuteri*-derived probiotic nano-formulation might be a promising treatment option for RAS.

## Introduction

Recurrent aphthous stomatitis (RAS) is a common type of oral ulcer that can occur in the general population, with a reported prevalence range of 0.7–50% [1]. It is more frequently observed in females and typically affects individuals between the ages of 7 to 44 years old [2, 3]. RAS ulcers can be classified into three forms based on their clinical presentation: minor, central, and herpetiform. Minor RAS lesions are the most common, representing over 80% of cases. They are characterized by small painful ulcers (5–10 mm) with a well-defined border that appears on non-keratinized mucosae such as the buccal mucosa, labial mucosa, tongue, soft palate, and pharynx [4, 5].

The exact cause of RAS remains unclear, but it is believed to involve multiple factors. Local factors such as minor trauma [6], altered microbiota, parafunctional habits, and systemic factors such as genetic susceptibility [7], trace element deficiencies (e.g., ferritin, zinc, and selenium) [8], chronic inflammatory gastrointestinal diseases, food allergy, hematologic conditions, and systemic medications (e.g., captopril, phenobarbital, diclofenac, and piroxicam) have been implicated in previous studies [9]. No specific curative medicine is available due to the unknown etiology of RAS. Therefore, various topical and systemic palliative drugs and pain relief methods have been used, including antiseptics, analgesics, steroids, antibiotics, and immunosuppressive drugs [10–14]. Topical coating agents are generally preferred for their effectiveness and safety, especially for mild to moderate cases [15]. Applying topical nano-formulations as efficient drug delivery to the lesions has also captured much attention [16–18].

In recent years, there has been a shift toward minimally invasive protocols for maintaining a balanced oral microbiota. These protocols include the use of probiotics and Para probiotics to rebalance oral flora, glycine and erythritol-based powders to target specific bacteria, and ozone therapy for its antibacterial and healing properties [19].

Probiotics are highly reproducible living organisms that can protect the mucosa from harmful microorganisms and have shown effectiveness in improving oral health in basic and clinical studies [20–22]. *Lactobacillus reuteri*, a probiotic, is naturally found in the gastrointestinal tract and has been studied for its potential benefits in oral health, including preventing halitosis, candidiasis, periodontitis, and caries. *Lactobacillus reuteri* can enhance oral immunity through two mechanisms: the production of reutroin, an antimicrobial compound that inhibits various opportunistic microorganisms, and the inhibition of TNF alpha and proinflammatory cytokines production [23, 24].

Although numerous studies have been conducted on the systemic effects of probiotics in treating oral lesions [25–28], there needs to be more research evaluating the therapeutic efficacy of probiotic nano-formulations on oral recurrent aphthous stomatitis. This study aimed to assess the therapeutic effects of a mixture of chitosan nanogel with a probiotic drug derived from *Lactobacillus reuteri* on recurrent aphthous lesions.

## Method and materials

### Materials

Chitosan Nanogel was purchased from Katokichi Co., Japan. *Lactobacillus reuteri* was generously donated by the Pasteur drug bank of Tehran.

### Participants

This randomized controlled trial was conducted at the School of Dentistry of Shiraz University of Medical Sciences, involving 60 adult patients (above 18 years old) diagnosed with minor aphthous lesions by two board-certified oral medicine specialists using the WHO index. This study complied with the ethical principles outlined by the Ethical Committee of Shiraz University of Medical Sciences, with approval granted under the ethical code IR.SUMS.DENTAL.REC.1398.103 and clinical trial code IRCT20110428006322N2 (date of registration: 17/10/2019). Written informed consent was obtained from all participants before their inclusion in the study.

Inclusion criteria included patients between 18 and 50 years old, without any systemic medication use in the past six months, presence of 1 to 3 simultaneous lesions, no more than one day since the appearance of the lesion, and no history of rheumatologic, gastrointestinal, renal diseases, or iron-deficiency anemia. Exclusion criteria were pregnancy and lack of cooperation. Participants were asked to abstain from consuming sources of probiotics, including yogurt and dietary supplements, throughout the duration of the study.

Using stratified block randomization, participants were randomly divided into two groups, the control group, and the probiotic group. The study was double-blinded, with neither the examiner nor the patients aware of the medication. The medication was distributed to each patient by an assistant according to the randomization blocks, and the assistant also provided instructions on how to use the assigned medication.

### Sample size and randomization

Based on previous studies, a sample size of 60 participants was determined to be sufficient to meet the power of 80% with a significance level of 0.05.

The patients were randomly allocated to two groups using a stratified block randomization method: the control and probiotic groups. The randomization was conducted with a block size of 4; each block contained four patients. The randomization process was carried out by a dental assistant who was not involved in the assessments or treatments to maintain blinding of the participants and examiners throughout the study.

### Drug preparation

The control group took routine palliative care. They were given an oral rinse, a mixture of 60 ml diphenhydramine, and 60 ml aluminum mg (ADIGEL-S). The control group was told to gargle the oral rinse told three times a day, for 3–4 min every time.

The probiotic group administered a topical Chitosan Nanogel/Probiotic mixture (CNP) thrice daily. They were told not to eat for at least 30 min after applying the oral, topical mixture. The *Lactobacillus reuteri* probiotic suspension was prepared by cultivating *Lactobacillus reuteri* in MRS (DeMan, Rogosa, Sharpe) broth at 37 °C for 48 h. The optical density of the culture was measured at a wavelength of 600 nm using a bio-photometer. When the density reached 0.8 (equivalent to the presence of 1 × 109 bacteria), the culture was centrifuged at 4000 rpm for 20 min. The pellet containing the bacteria was resuspended in an appropriate volume of phosphate-buffered saline (PBS), and the supernatant was discarded. All processes were conducted under aseptic conditions in a hood.

A 10 ml aliquot of the bacterial suspension was removed and added to the MRS agar culture medium, which was then incubated at 37 °C for 24 h to confirm the viability of the *Lactobacillus reuteri*.

*Lactobacillus reuteri* suspension was concentrated using an ultra-filtration kit with a 30,000 molecular weight cut-off membrane to retain the probiotic bacteria while removing excess buffer. The concentrated probiotic suspension was mixed with the chitosan nanogel at a 1% (v/v) concentration. The mixture was stirred thoroughly using a magnetic stirrer for 24 h to ensure homogeneous distribution of the probiotic within the nanogel. The prepared Chitosan Nanogel/Probiotic mixture (CNP) was stored in containers at four °C for patient use.

### Evaluation and indices

The data for this study was obtained through clinical examination and observation. Patients were assessed for pain intensity and lesion size in four consecutive sessions, namely before, on day 3, day 5, and day seven after the treatment. Lesion size was measured using a calibrated caliper and reported in millimeters. Pain severity was assessed using the Visual Analog Scale (VAS).

### Statistical analysis

The data were analyzed using SPSS software version 26. Mean ± standard deviation (SD) was used to report the descriptive statistics of the data. Wilcoxon test was utilized to compare the Visual Analog Scale (VAS) scores at different time points (0, day 3, day 5, and day 7). In contrast, a repeated measurement test was employed to compare the lesion size across the same time points. Additionally, the Mann-Whitney test was used to compare each session’s intervention scores. A significance level of *P* < 0.05 was considered statistically significant.

## Results

The study comprised two groups, each consisting of 30 participants. Throughout the course of this trial, no patients withdrew or dropped out from the study. None of the participants reported any adverse effects or disturbances during the interventions. There were 18 females in the control group and 16 in the probiotic group. The distribution of males and females did not show a statistically significant difference according to the chi-square test (P = 0.30). The age of the patients ranged from 20 to 48 years, with a mean age of 27.82 ± 6.79 in the probiotic group and 28.62 ± 6.35 in the control group. There was no statistically significant difference in the mean age between the two groups according to the independence test (P = 0.71). The VAS and lesion size results in the two groups did not show any statistically significant difference, as shown in Tables 1 and 2.

The assessment conducted over four consecutive sessions revealed favorable healing of lesions in both the control and probiotic groups. Table 1 presents the mean size of the lesions, and Table 2 displays the pain intensity assessed using the VAS in both groups. Analysis using the general linear model and repeated measures test, considering the measurement of quantitative variables across multiple sessions, demonstrated that both groups exhibited statistically significant changes in lesion size and VAS over time. Additionally, a significant difference was observed in mean lesion size and VAS between each session and its adjacent sessions.

**Table 1:** Mean lesion size over sessions in control and probiotic group

	Before intervention	Day 3	Day 5	Day 7
Control group	7.14 ± 2.12	5.26 ± 1.69	3.26 ± 1.55	1.20 ± 0.81
Probiotic group	7.88 ± 1.44	4.44 ± 1.47	1.32 ± 1.18	0.47 ± 0.60
*P*-value	0.24	0.00	0.00	0.01

**Table 2:** Mean VAS over sessions in control and probiotic group

	Before intervention	Day 3	Day 5	Day 7
Control group	6.64 ± 1.41	5.00 ± 1.06	2.47 ± 0.80	0.82 ± 0.80
Probiotic group	7.17 ± 1.42	3.70 ± 1.57	1.55 ± 1.17	0.5 ± 0.21
*P*-value	0.28	0.00	0.00	0.04

**Fig. 1:**
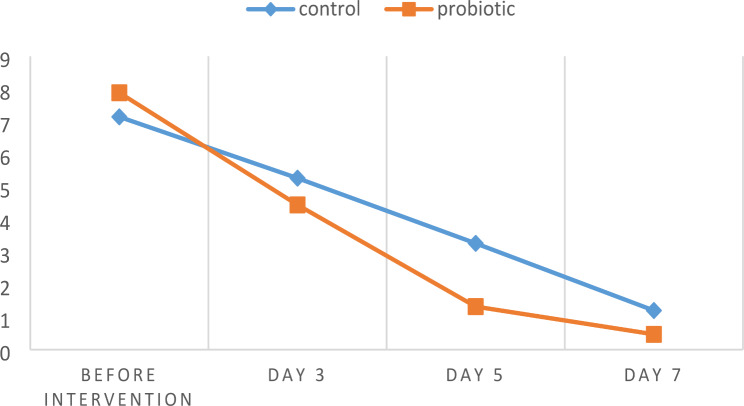
Mean lesion size over sessions in control and probiotic group

**Fig. 2:**
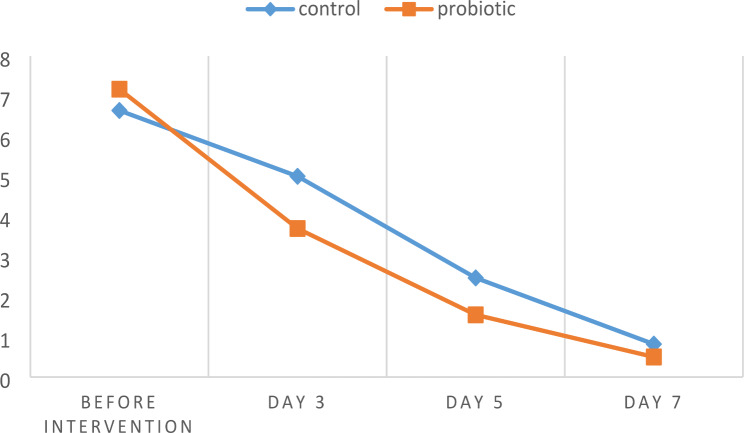
Mean VAS over sessions in control and probiotic group

The study’s findings revealed that the probiotic group exhibited a mean reduction of 7.41 ± 1.79 millimeters in lesion size and a mean reduction of 6.54 ± 1.56 scores in pain intensity at the end of the study. The control group exhibited a reduction of 5.94 ± 1.59 millimeters in lesion size and a reduction of 5.82 ± 1.66 scores in pain intensity. (Figs. 1 and 2) In terms of Mauchly’s test of sphericity, and the independent t-test analysis, the reductions in lesion size and pain severity were statistically significant in the probiotic group compared to the control group.

## Discussion

Given the prevalence of recurrent aphthous stomatitis (RAS), we aimed to investigate the efficacy of a topical *Lactobacillus reuteri-*derived probiotic nano-formulation in reducing lesion size and pain intensity in patients with RAS.

The study results showed that the probiotic drug derived from *Lactobacillus reuteri* exhibited a statistically significant reduction in lesion size and pain intensity compared to the control group. The probiotic group demonstrated faster and more substantial healing than the control group, indicating that the probiotic nano-formulation was more effective than common analgesic care. Notably, there was no significant difference in the effect of the probiotic nano-formulation between genders.

A recent hypothesis suggests it may be due to a local immune system defect triggered by oral bacteria. Studies show differences in oral microbiota between RAS patients and healthy individuals, with imbalances in specific bacteria potentially contributing to RAS symptoms such as ulcers, delayed healing, and severe pain [29–31]. The findings of this study could be attributed to the potential immunomodulatory and anti-inflammatory properties of *Lactobacillus reuteri* [32]. Probiotics are believed to modulate the immune system, correct dysbiosis, strengthen mucosal barriers and regulate inflammatory responses [33–36]. They also improve the integrity of the mucosal epithelium by inhibiting the production of pro-inflammatory cytokines, such as TNF-, and subsequently, decrease epithelial permeability [33]. The modulation of the oral microbiota by the probiotic formulation may have reduced pain and lesion size in patients with RAS. Using probiotics as a nano-formulation may enhance their stability, bioavailability, and effectiveness in delivering beneficial bacteria to the oral cavity, thereby exerting their therapeutic effects on RAS [37].

This study’s findings agree with previous studies that reported probiotics’ beneficial effects in managing RAS. Pedersen et al. showed that a probiotic lozenge containing *Lactobacillus reuteri* improved the Ulcer Severity Score and reduced oral pain related to RAS over a 3-month [38]. However, the improvement was not statistically significant compared to the placebo group [38]. Nirmala et al. showed considerable improvement in erythema, pain reduction, decreased oral thrush, and burning sensation in the mouth following topical application of *Bacillus Clausii* probiotics can be used as an adjuvant in treating recurrent aphthous ulcer and oral candidiasis [27]. These studies and the present study suggest that probiotics may be a promising therapeutic option for managing RAS.

However, it is worth noting that some previous studies have reported conflicting results. For example, a systematic review and meta-analysis conducted by Cheng et al. found probiotics effective in relieving oral pain but ineffective in reducing ulcer severity [39]. Aggour et al. reported significant differences in pain reduction and insignificant ulcer size reduction following applying *Lactobacillus acidophilus* probiotic lozenges compared to the control group [40]. These discrepancies in findings could be attributed to differences in study design, probiotic strains, formulations used, dosages, and study populations.

Nanofibers, nanoparticles, and nanostructured materials have shown promise for probiotic delivery due to their efficient encapsulation, site-specific release, stability during manufacturing and storage, biocompatibility and controlled drug release, and improved viability [37]. Overall, nanomaterials-based formulations can enhance the therapeutic effects of probiotic products [37].

Despite the promising results of this study, some limitations need to be considered in future studies. The sample was small, and the follow-up period was limited to four sessions. The study did not investigate the mechanisms underlying the beneficial effects of the probiotic nano-formulation via in-vitro tests. Our analysis also lacked a separate chitosan nanogel group so that we could investigate the possible positive effect of chitosan as an anti-bacterial and anti-inflammatory polymer on the study result.

In light of the encouraging results from this study, it is worth considering the potential implications for future research and clinical applications. The concept of para probiotics, probiotics, and postbiotics presents exciting opportunities in the field of oral health. Probiotics are effective in promoting various health benefits, but concerns about their safety have emerged, especially when administered to vulnerable individuals like the elderly and those with weakened immune systems. In response to these concerns, non-viable probiotic products known as para probiotics and postbiotics have been introduced. Para probiotics are inactivated microbial cells that offer health benefits without posing health risks. They can regulate the immune system, combat pathogens, and exhibit anti-inflammatory, antiproliferative, and antioxidant properties [19, 41]. Furthermore, future studies could delve deeper into the specific mechanisms underlying the beneficial effects of probiotics and para probiotics. In-vitro tests and mechanistic research are warranted to elucidate the precise interactions between these microbial formulations and the oral environment, shedding light on the pathways through which they exert their therapeutic effects.

## Conclusions

Considering the limitations, the findings of this study contribute to the growing body of evidence on the potential benefits of probiotics in managing RAS and highlight the importance of further research in this field. *Lactobacillus reuteri*-derived probiotic nano-formulation might be a promising treatment option for RAS. Moreover, the study emphasizes the need for further research to explore proactive actions with para probiotics, probiotics, and postbiotics which present exciting opportunities to enhance oral health care.

## Data Availability

The datasets generated during and analyzed during the current study are available from the corresponding author upon reasonable request.
